# Draft Genome Sequence of Burkholderia gladioli Coa14, a Bacterium with Petroleum Bioremediation Potential Isolated from Coari Lake, Amazonas, Brazil

**DOI:** 10.1128/genomeA.00301-18

**Published:** 2018-04-19

**Authors:** Eraldo Ferreira Lopes, Josemar Gurgel Da Costa, Ivan Rodrigo Wolf, José Paulo de Araújo Lima, Spartaco Astolfi-Filho

**Affiliations:** a Institute of Health and Biotechnology, Federal University of Amazonas, Coari, Amazonas, Brazil; b College of Agronomic Sciences of Botucatu, State University of São Paulo Júlio de Mesquita Filho, Botucatu, São Paulo, Brazil; c Institute of Biological Sciences, Federal University of Amazonas, Manaus, Amazonas, Brazil

## Abstract

Burkholderia gladioli Coa14 is a bacterium isolated from water collected from Coari Lake (Amazonas, Brazil) that shows a capacity for survival in a medium containing only oil as a carbon source. Here, we report its draft genome sequence, highlighting some genes involved with petroleum derivative degradation.

## GENOME ANNOUNCEMENT

Burkholderia gladioli is a mobile, Gram-negative, and catalase-positive bacterium that belongs to the Burkholderia cepacia complex. It has symbiotic fungal activities and is able to produce disease in plants and in immunosuppressed humans (1). This genus has biotechnology applications, including biological control, biostimulation, and bioremediation (2, 3). Thus far, there are different lineages of B. gladioli associated with bioremediation (4–6).

This lineage was isolated through culture enrichment (7) by means of water collected from Coari Lake, which belongs to the city of Coari (Amazonas, Brazil), along the Petrobrás SA Rio Solimões Oil Pipeline (ORSE-I). Isolation was conducted using a minimal mineral medium containing crude oil from the Urucú oil-producing province, in Coari’s urban area. Afterwards, the B. gladioli sample was grown for 21 days in Bushnell Haas medium containing crude oil as the only carbon source.

The cells were isolated in order to extract DNA using the phenol-chloroform method. Extraction quality and concentration were evaluated by NanoDrop and Qubit instruments, respectively. There were 2 rounds of DNA sequencing with an Illumina QTE HiSeq instrument, generating 10,855,172 paired-end reads of 250 nucleotides (nt).

The reads were evaluated for quality and had their sequencing adapters removed using Trimmomatic v0.32 (8). The *de novo* assembly was made with MIRA v4.0-1 (9) and CAP3 (10), which generated 23 contigs and a draft genome of 8.4 Mb. The *N*_50_ value obtained was 574,923 bp, the genome was 95.06% of the B. gladioli ATCC 10248 reference genome size (11), and the G+C content estimated for the draft genome sequence was 68.04%. Prokka 1.12 (12) and the Rapid Annotations using Subsystem Technology (RAST) Web server (13) identified the presence of 7,155 coding sequence (CDS) regions and 7,540 genes, 76 of them for tRNA and 1 for transfer-messenger RNA (tmRNA).

Gas chromatography-mass spectrometry (GC-MS) analysis showed that this lineage is capable of reducing 40.17% of the total *n*-alkanes in Urucú crude oil. Annotations revealed the presence of all the genes in the *n*-alkane degradation pathway described by Koshlaf and Ball in 2016 (14). This pathway begins with the alkane 1-monooxygenase and converges with the fatty acid oxidation pathway.

Other important enzymes for petroleum derivative degradation pathways were noted, including 4,5-dihydroxyphthalate decarboxylase (15) and catechol 1,2-dioxygenase (16), both present as reaction initiators for opening aromatic rings during the initial degradation stages of polycyclic aromatic hydrocarbons present in the oil. Annotations also revealed the presence of genes for alpha and beta chains of protocatechuate 3,4-dioxygenase (17, 18) and homogentisate 1,2-dioxygenase (19).

This information regarding Burkholderia gladioli Coa14 may help us to better understand the mechanisms used for its survival in environments impacted by oil spills through its ability to degrade petroleum components. Once these mechanisms are understood, they could be explored in biotechnology processes applied to environmental protection, primarily in areas of substantial associated biodiversity, such as Amazonia.

### Accession number(s).

This whole-genome shotgun project has been deposited in DDBJ/ENA/GenBank under the accession number PQII00000000. The version described in this paper is version PQII01000000.
